# Clinical and radiological hip parameters do not precede, but develop simultaneously with cam morphology: a 5-year follow-up study

**DOI:** 10.1007/s00167-020-06282-0

**Published:** 2020-10-01

**Authors:** P. van Klij, M. P. Heijboer, A. Z. Ginai, J. A. N. Verhaar, J. H. Waarsing, R. Agricola

**Affiliations:** 1 Department of Orthopaedic Surgery, Erasmus MC, University Medical Center Rotterdam, PO Box 2040, 3000 CA Rotterdam, The Netherlands; 2 Department of Radiology, Erasmus Medical Center Rotterdam, Rotterdam, The Netherlands

**Keywords:** Hip, Stress, Development, Growth plate, Range of motion

## Abstract

**Purpose:**

The aim of this study was to (1) investigate whether radiographic and clinical parameters, which influence how stresses during sporting activities act on the proximal femur, are associated with cam morphology or (2) precede cam morphology development.

**Methods:**

Young male football players participated at baseline (*n* = 89, 12–19 years of age), 2.5-year (*n* = 63) and 5-year follow-up (*n* = 49). Standardized anteroposterior pelvic and frog-leg lateral radiographs were obtained at each time-point. Cam morphology was quantified by an alpha angle ≥ 60°, and large cam morphology ≥ 78°. The neck–shaft angle (NSA), epiphyseal extension (EE), lateral center–edge angle (LCEA) and hip internal rotation (IR) were also measured. Cross-sectional associations between NSA, EE, LCEA and IR and (large) cam morphology were studied at all time-points. To study whether these variables preceded cam morphology development, hips without cam morphology at baseline were studied prospectively.

**Results:**

A lower NSA, a higher EE and limited IR were consistently associated with cam morphology at all three time-points. These differences were more pronounced in hips with large cam morphology. No association between cam morphology and the LCEA was found. None of the parameters studied preceded cam morphology development.

**Conclusion:**

Cam morphology developed simultaneously with a varus orientation, growth plate extension towards the femoral neck and limited hip internal rotation. These parameters did not precede cam morphology development. The hip parameters studied cannot be used to identify individuals at risk of developing cam morphology.

**Level of Evidence:**

Level II.

**Electronic supplementary material:**

The online version of this article (10.1007/s00167-020-06282-0) contains supplementary material, which is available to authorized users.

## Introduction

Cam morphology is extra bone formation on the anterolateral head–neck junction of the proximal femur and is associated with an increased risk of developing hip osteoarthritis (OA) [2, 16, 20, 26, 30].

The etiology of cam morphology has still not been fully understood. Several studies have found that it forms during growth [22, 28, 31], is slightly more prevalent in males (15–25%) than in females (5–15%) [9, 11, 24], and is more common in professional athletes [1, 3, 22, 31]. A finite element study showed that the stress distribution resulting from different loading patterns on the immature and growing proximal femur influenced the trigger for bone formation at the location where cam morphology normally develops [25]. Cam morphology development also depends on growth plate orientation, when the growth plate extends toward the neck. This results in a stimulus for bone formation at the anterolateral head–neck junction. Not only the orientation of the growth plate, but also varus/valgus orientation might influence the stress distribution through the growing proximal femur and thereby the risk of cam morphology development [6, 25]. Since the development of the growing hip is an interplay between the proximal femur and the acetabulum, cam morphology development might also be influenced by acetabular coverage.

Clinically, cross-sectional studies have shown associations between lower neck–shaft angles (NSA) [13] and an extended growth plate towards the femoral neck [22] and cam morphology. The link between acetabular coverage and cam morphology development has not been examined. The relationship between cam morphology and the amount of hip joint internal rotation is also unclear [8, 12, 15]. Cam morphology might cause abutment between the proximal femur and acetabulum, thereby limiting hip internal rotation. Palmer et al. [22] showed that an osseous cam morphology might be preceded by a cartilaginous bump, which might even lead to limited internal rotation before osseous cam morphology is present.

To date, no longitudinal studies on the relationship between the above-mentioned parameters and cam morphology are available. It is therefore unknown if these hip parameters develop simultaneously, or whether they actually precede cam morphology development, and therefore are a cause of cam morphology development. If the latter was true, one would be able to identify which adolescents are at highest risk of developing cam morphology before its actual presence, which allows a selection for preventative measures.

The study aims were (1) to investigate whether radiographic (NSA, EE, LCEA) and clinical (internal rotation) factors were associated with cam morphology presence and/or (2) whether these factors preceded cam morphology development. The hypothesis was that the hip parameters examined were associated with cam morphology presence and size, but that they did not precede the development of cam morphology. This might provide new insights into which radiographic or clinical factors could predict those who are susceptible to developing cam morphology.

## Materials and methods

**Fig. 1:**
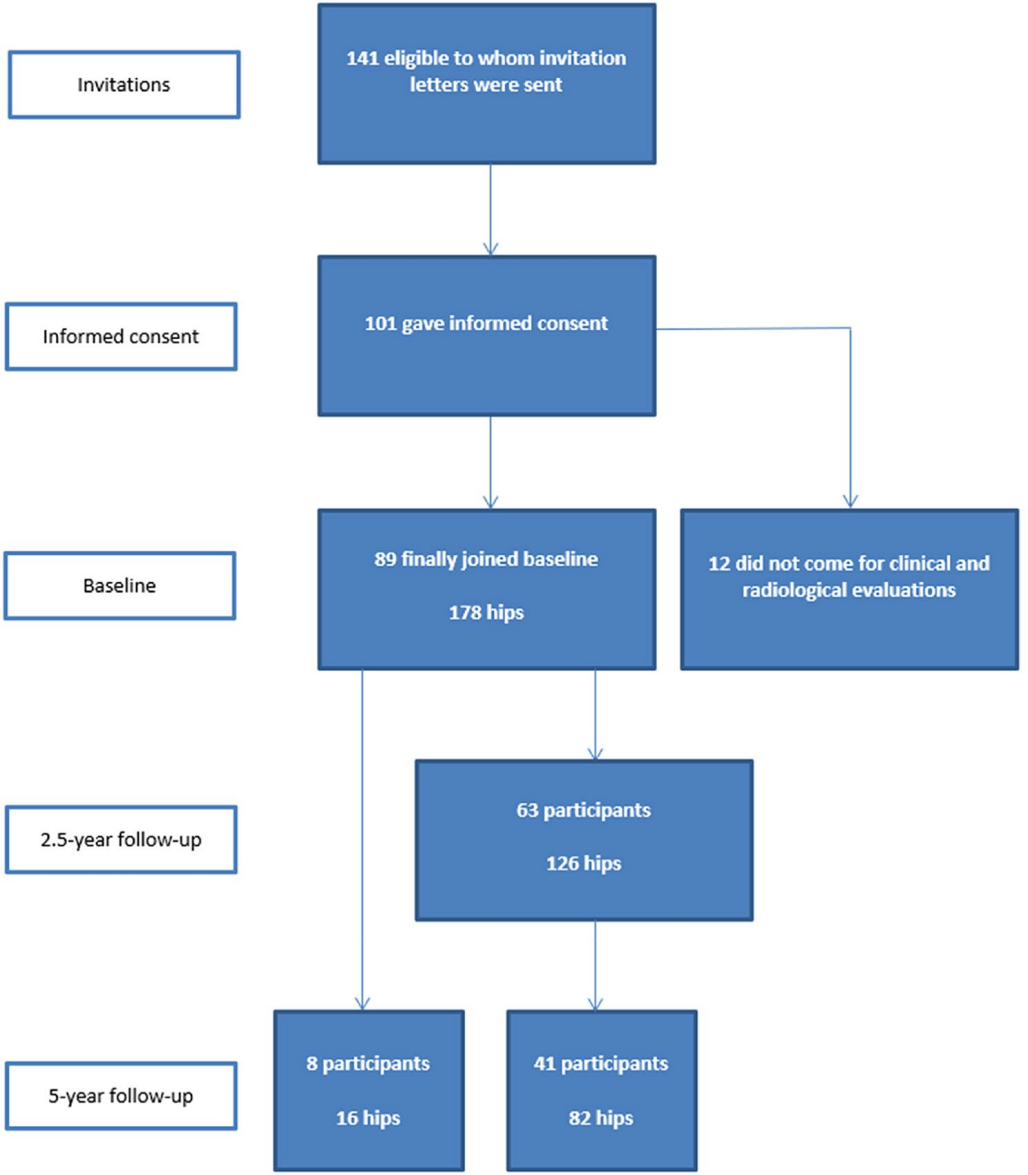
Flowchart of all participants at baseline, 2.5-year follow-up and 5-year follow-up. The 8 participants at 5-year follow-up, were participants who did not attend at 2.5-year follow-up

The Medical Ethical Committee of the Erasmus Medical Center (Rotterdam, The Netherlands) approved this study (IRB: NL28614.078.09). Written consent was obtained from all participants. For participants aged under 18 years, written consent from at least one parent was also obtained. The inclusion and exclusion criteria for this study have been described previously [1, 3]. Adolescent male football players who played in selection teams of Feyenoord football club in Rotterdam (The Netherlands) were included. Exclusion criteria were any known hip disorder. At baseline, information letters were sent to all eligible asymptomatic athletes (*n* = 141), of whom 101 gave informed consent and 89 (12–19 years of age) joined this study at baseline. At 2.5-year follow-up, 63 participants were included and at 5-year follow-up, 49 participants (mean age, 20.5 ± 2.2 years) (Fig. 1). The 5-year follow-up was performed between June and October 2015.

### Radiographs

A standardized radiographic protocol was used at baseline, 2.5-year and 5-year follow-up, which has been described previously [1, 3]. In summary, three radiographs of the hip were obtained: a standardized supine anteroposterior (AP) radiograph of the pelvis and a frog-leg lateral radiograph of each hip.

### Cam morphology presence, size and development

**Fig. 2:**
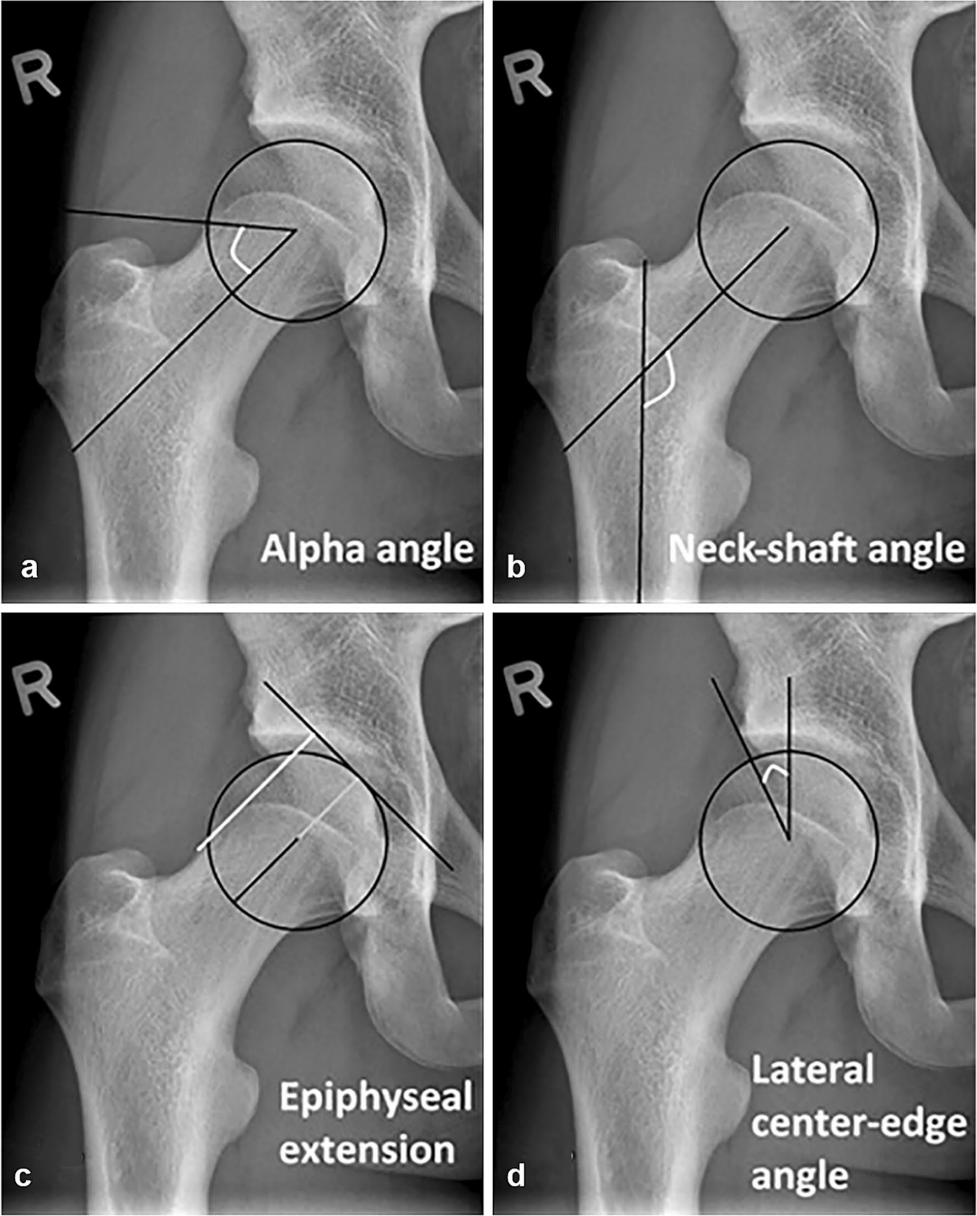
The radiographic measurements of the same right hip. **a** The alpha angle (white angle) is measured by drawing a best fitting circle around the femoral head and a line through the center of the neck and the center of the femoral head. From the center of the femoral head, a second line is drawn to the point where the superior surface of the head-neck junction departs from the circle the first time. The angle formed by these two lines is the alpha angle. **b** The NSA neck-shaft angle (NSA, (white angle) is the angle determined by a line through the middle of the femoral shaft and a line through the middle of the femoral head and neck, with a higher value indicating a valgus orientation and a lower value a varus orientation. **c** The epiphyseal extension (EE) was measured as described by Siebenrock et al. [29]. First, a perpendicular line to the line through the middle of the femoral head and neck is drawn. From this line, again a perpendicular line (white line) is drawn to the lateral endpoint of the growth plate. The distance of this line is divided by the femoral head radius (grey line), which results in the EE. **d** The lateral center-edge angle (LCEA), also known as the Wiberg angle [32], measures the amount of lateral acetabular coverage relative to the femoral head. It is calculated by a vertical line from the middle of the femoral head, which is perpendicular to the horizontal line connecting the two superolateral portions of the obturator foramen, to correct for coronal balance. Then, the second line departs also from the middle of the femoral head towards the most lateral point of the acetabulum

The shape of the proximal femur was outlined by a manually positioned set of points on predefined anatomical landmarks, using Statistical Shape Modelling software (ASM tool kit, Manchester University, Manchester, UK) (Fig. 2a**)**. Cam morphology was quantified using the alpha angle. The alpha angle was calculated automatically by using MATLAB v7.1.0 (MathWorks Inc, Natick, Massachusetts, USA) from the set of points on the AP and frog-leg lateral radiographs, placed by one observer (PvK), for all time-points [1, 3]. Cam morphology presence was defined as an alpha angle ≥ 60° [5], in either the AP or frog-leg lateral radiograph of each hip. Large cam morphology was defined as an alpha angle ≥ 78° in either view [5]. When a hip had an alpha angle ≥ 60° at a certain point, we defined this hip as having cam morphology at the subsequent follow-up time-points as well.

Cam morphology development was defined as a change in alpha angle from < 60° to ≥ 60°. In order to study if the NSA, EE, LCEA and internal rotation preceded cam morphology development, we only analyzed hips without cam morphology at baseline and with at least one follow-up time-point available. If two follow-up time-points were available (i.e., both 2.5-year and 5-year follow-up), the last time-point was used for analysis. Of these hips, the baseline parameters NSA, EE, LCEA and internal rotation were compared between hips that did and did not develop cam morphology in time. The intra-class correlation coefficient (ICC) of the alpha angle for inter-observer reliability was 0.73 and for intra-observer reliability 0.85–0.99 [2].

### Growth plate status

The proximal femoral growth plate was scored based on consensus by an experienced musculoskeletal radiologist (AZG) and an experienced orthopaedic surgeon (MPH). If only part of the growth plate remained open in any radiographic view, that growth plate was scored as open. If the full growth plate was totally fused and visible as a sclerotic line it was scored as closed. A kappa of 0.94 for intra-observer reliability was observed.

### Radiographic and clinical parameters

The radiographic independent parameters NSA, EE and LCEA were all measured on AP radiographs. The measurement methods are described in Fig. 2. The amount of hip internal rotation was determined by physical examination [1]. While maintained in neutral rotation, the first resistance/end feel during passive internal rotation was measured in supine position on a flat examination table with a goniometer. Internal rotation was measured with 90° of flexion in the hip joint. The inter-observer variability for NSA, EE and LCEA was determined by scoring 10 random radiographs by 2 persons (PvK and RA) and was 0.97 for NSA, 0.87 for EE and 0.94 for LCEA. The intra-observer variability was 0.98 for NSA, 0.86 for EE and 0.99 for LCEA.

### Statistical analysis

Differences in characteristics between participants and dropouts were tested by an independent samples t-test. Cam morphology presence and size was described per hip. The cross-sectional association between the variables NSA, EE, LCEA and internal rotation and cam morphology presence and size were analyzed and calculated by a logistic regression at all three time-points. This resulted in the analysis of 178 hips at baseline, 126 hips at 2.5-year follow-up and 98 hips at 5-year follow-up. By using logistic regression in a ‘Generalized Estimated Equations’ (GEE) model, we could model the correlations that existed within a person regarding side. The analyzes were corrected for age and body mass index (BMI). For the associations between NSA, LCEA, internal rotation and cam morphology, the odds ratio (OR) and 95% confidence interval are presented per degree difference. For the EE, the OR and 95% confidence interval are presented for increments of 0.01. The NSA, EE, LCEA and internal rotation were studied in a longitudinal design to observe if there were any differences in these values at baseline between football players that did or did not develop cam morphology, using a GEE model with logistic links function, adjusted for age and BMI. The unadjusted data are presented in a sensitivity analysis (Supplemental Table 1). SPSS25.0 (Windows) was used for statistical evaluation.

## Results

### Participant characteristics

The demographic data of all participants is presented in Table 1 [31]. The mean follow-up was 5.3 ± 0.1 years (range 5.0–5.6 years). No significant differences in demographic baseline characteristics were observed between the 5-year follow-up participants and drop-outs (Table 2) [31]. At 5-year follow-up, all participants still played football, 28 of 49 (57%) at a professional level, 21 of 49 (43%) as an amateur. The prevalence of cam morphology and large cam morphology is presented in Table 1.

**Table 1:** Demographic data and cam morphology prevalence at baseline, 2.5-year follow-up and 5-year follow-up

Participant characteristics	Baseline (*n* = 89)	2.5-year follow-up (*n* = 63)	5-year follow-up (*n* = 49)
Age, year	15.2 ± 2.0	17.3 ± 2.0	20.5 ± 2.2
Weight, kg	59.4 ± 13.8^	68.4 ± 11.1^#^	73.8 ± 7.9
Height, cm	170.3 ± 12.2^	177.44 ± 8.0^#^	180.3 ± 6.6
Body mass index, kg/m^2^	20.1 ± 2.3^	21.6 ± 2.2^#^	22.7 ± 1.6
Football experience, year	9.0 ± 2.54^	11.1 ± 2.54^#^	14.3 ± 2.68
Training intensity, h/week	8.0 ± 1.8^	8.7 ± 1.9^##^	9.3 ± 2.9
*Cam morphology prevalence per hip*	178 hips	126 hips	98 hips
Cam	87 (48.9%)	86 (68.3%)	78 (79.6%)
Large cam	24 (13.5%)	25 (19.8%)	25 (25.5%)

Due to missing data, data of *n* = 87 (^), *n* = 58 (#) and *n* = 57 (##) are presented

Values are expressed as mean ± standard deviation, with *n* = participants

**Table 2:** Demographic baseline data of 5-year follow-up participants and drop-outs

Participant characteristics	Baseline (*n* = 49) 5-year follow-up participants	Baseline (*n* = 40) 5-year follow-up drop-outs	*P* value
Age, year	15.2 ± 2.1	15.3 ± 1.8	n.s.
Weight, kg	58.54 ± 14.7	60.4 ± 12.6^#^	n.s.
Height, cm	169.4 ± 13.2	171.5 ± 10.7^#^	n.s.
Body mass index, kg/m^2^	20.0 ± 2.3	20.3 ± 2.2^#^	n.s.
Football experience, year	8.84 ± 2.7	9.1 ± 2.4^#^	n.s.
Training intensity, h/week	7.9 ± 1.6	8.1 ± 2.0^#^	n.s.
*Radiographic and clinical parameters*	*n* = 98 hips	*n* = 80 hips	
Cam morphology prevalence, %	48.0	50.0	n.s.
NSA	131.2° ± 5.3°	131.8° ± 5.4°	n.s.
EE	1.49 ± 0.19	1.58 ± 0.19	0.004
LCEA	26.9° ± 6.1°	27.9° ± 7.3°	n.s.
Internal rotation	26° ± 8°	25° ± 9°	n.s.

Values are expressed as mean ± standard deviation, with *n* = participants for baseline characteristics and *n* = hips for independent and dependent parameters

Due to missing data, data of *n* = 38 (^#^) are presented

*NSA* neck-shaft angle, *EE* epiphyseal extension, *LCEA* lateral center-edge angle, *n.s.* non-significant

### Cross-sectional associated parameters

#### Neck-shaft angle (NSA)

**Fig. 3:**
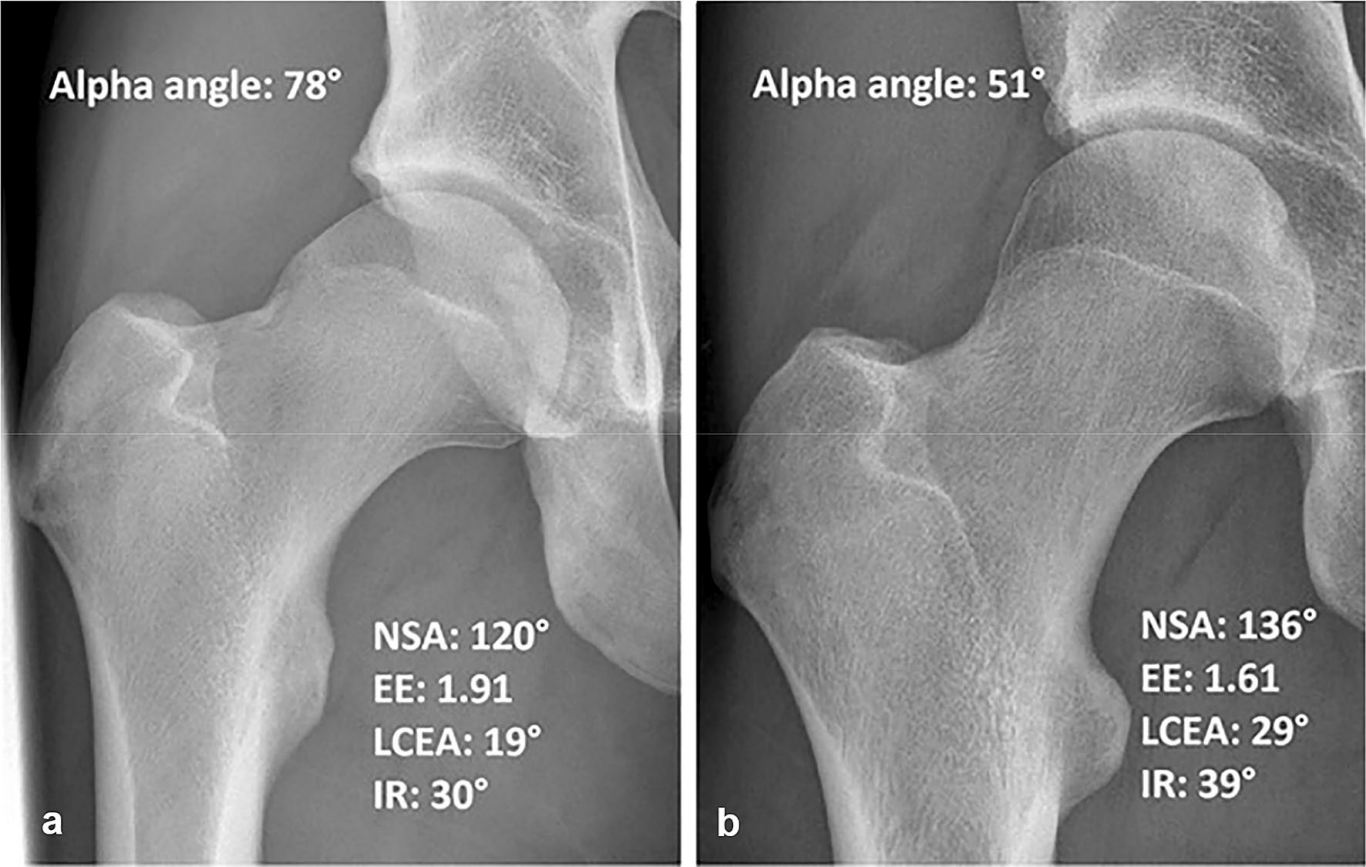
An example of two hips of different participants with closed growth plates, both at 5-year follow-up. **a** A typical hip with cam morphology, varus orientation and an extended growth plate towards the neck. **b** A hip without cam morphology with a more valgus orientation and without an extension of the growth plate towards the femoral neck. *EE* epiphyseal extension, *IR* internal rotation, *LCEA* lateral center-edge angle, *NSA* neck-shaft angle

**Fig. 4:**
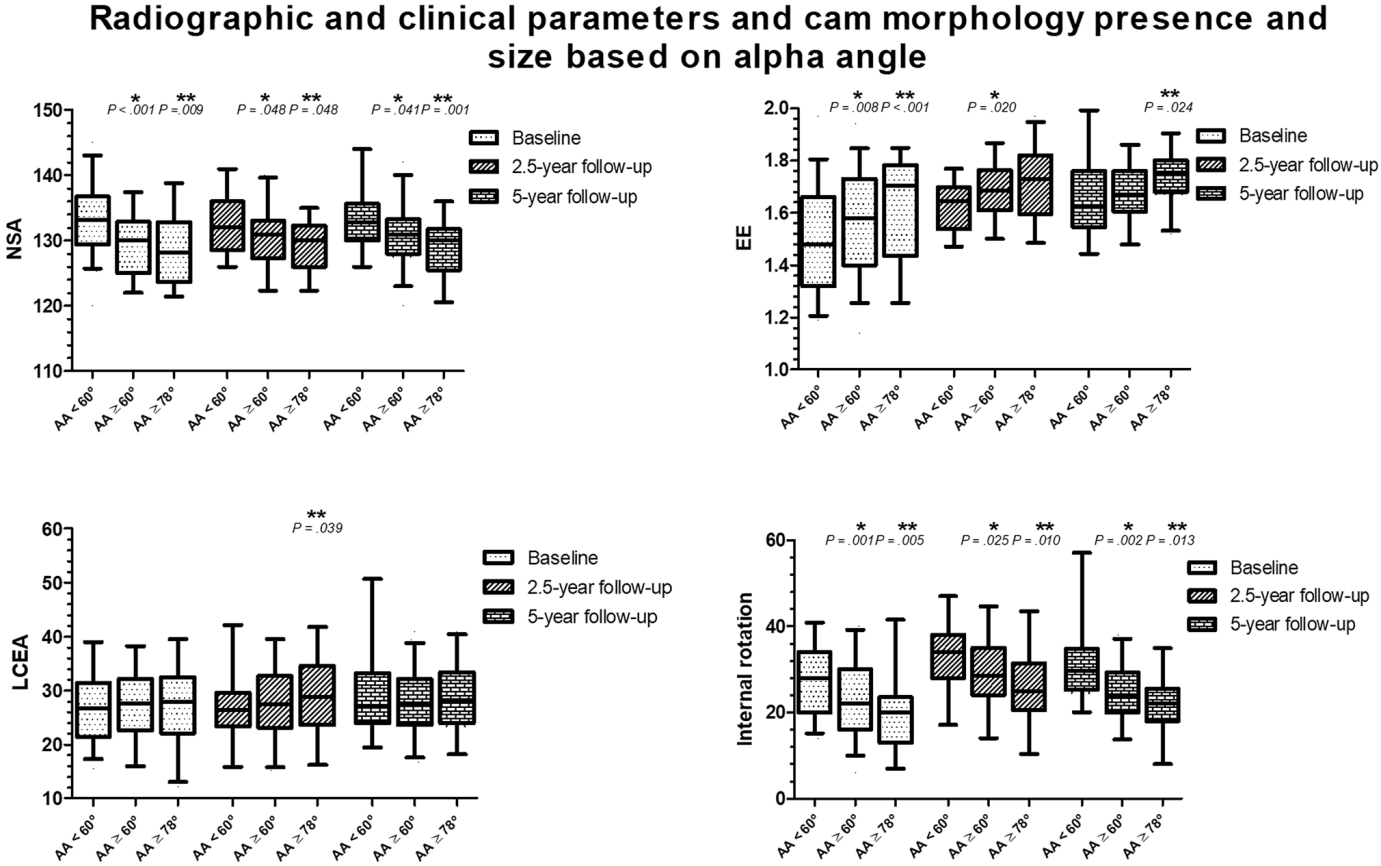
The associations between the NSA, EE, LCEA, hip internal rotation and cam morphology presence and size visualised in a boxplot. In these plots, the box with 25–75th percentile and median (horizontal line) are presented. The whiskers represent the 5–95th percentile. The associations are corrected for age and BMI. *Significant association between the parameter and cam morphology, compared to hips without cam morphology. **Significant association between the parameter and large cam morphology, compared to hips without large cam morphology

The NSA was significantly associated with both cam morphology and large cam morphology at all three time-points, compared to hips without cam morphology (Figs. 3, 4 and Supplemental Table 2/3/4).

#### Epiphyseal extension (EE)

EE was significantly associated with cam morphology presence at baseline and 2.5-year follow-up, and with large cam morphology at baseline and 5-year follow-up, when compared to hips without cam morphology (Figs. 3, 4 and Supplemental Table 2/3/4).

#### Lateral center-edge angle (LCEA)

No association between the LCEA and cam morphology was observed at any time-point. The LCEA was associated with large cam at 2.5-year follow-up (Figs. 3, 4 and Supplemental Table 2/3/4).

#### Internal rotation

The amount of internal rotation was associated with cam morphology presence and size at all time-points, compared to hips without cam morphology (Figs. 3, 4 and Supplemental Table 2/3/4).

### Preceding baseline parameters

Seventy-two hips had no cam morphology at baseline, and 55 of them an open growth plate (77%). During follow-up, 43 of 72 hips (60%) developed cam morphology, of which 37 had an open growth plate (86%) at baseline. The NSA, EE, LCEA and internal rotation were not significantly different between hips that did or did not develop cam morphology during follow-up. (Table 3)

**Table 3:** Cam morphology development during follow-up and baseline preceding parameters

	Development (*n* = 43)	No development (*n* = 29)	*P* value
NSA	133.60° ± 4.78°	133.18° ± 5.34°	n.s.
EE	1.41 ± 0.15	1.47 ± 0.19	n.s.
LCEA	26.56° ± 6.07°	26.25° ± 6.31°	n.s.
Internal rotation	28° ± 8°	28° ± 8°	n.s.

Values are expressed as mean ± standard deviation, with *n* = participants and all *P* values were corrected for age and BMI

*NSA* neck-shaft angle, *EE* epiphyseal extension, *LCEA* lateral center-edge angle, *n.s.* non-significant

## Discussion

The main finding of this study is that a lower NSA, higher EE and decreased hip internal rotation developed simultaneously with cam morphology. This suggests that certain biomechanical stresses on the growing hip that predispose to developing cam morphology can also lead to a more varus orientation and growth plate extension towards the neck. This process of proximal femoral anatomy development during growth occurs simultaneously. This is in keeping with a finite element study [25] which observed that loading conditions influences growth plate shape and cam morphology development. Interestingly, these factors did not precede cam morphology development. In other words, there does not seem to be a causative relationship between these factors and subsequent cam morphology development. These parameters cannot assist in the prediction of cam morphology development.

This study found that increasing varus orientation of the hip was associated with cam morphology presence and size. This corresponds with a recent study [13] of 33 professional ballet dancers and 33 age- and sex-matched athletes. They found a lower NSA in athletes than in ballet dancers (130.8° ± 4.7° vs 134.6° ± 4.6°). Interestingly, the athletes also had a higher cam morphology prevalence. Other studies also found an association between lower NSA and symptoms [10, 17–19]. This may imply that a lower NSA is not only associated with cam morphology but may also lead to more symptoms. A possible explanation is that cam morphology in varus hips might lead to premature contact with the acetabulum when compared to hips with cam morphology with a valgus orientation. This is in keeping with the CHECK prospective study [4], where hips with cam morphology and a varus orientation had higher risk of developing hip OA than hips without a varus position.

In this study, an increased EE was cross-sectionally associated with cam morphology, but did not precede cam morphology development. This is in line with Siebenrock et al. [29] who described an association between epiphyseal extension and cam morphology in a group of 15 participants with cam morphology compared with 15 controls. Three other studies also found a correlation between epiphyseal extension and the alpha angle [14, 22, 27]. There is no previous longitudinal study available on EE and the development of cam morphology. These clinical studies fit with a finite element study [25], which showed higher shear stresses (a trigger for bone formation) at the location where cam morphology develops when the growth plate extended towards the femoral neck. However, in the current study there was no evidence that an extended growth plate preceded cam morphology development. It is therefore probably a simultaneously occurring adaptive response to mechanical load applied to the growing hip.

The absence of a significant association between the LCEA and cam morphology corresponds with the previous findings of Anderson et al. [7]. Although there is an interplay between the acetabulum and proximal femur during growth, the lateral acetabular coverage apparently does not have an effect on developing cam morphology. However, the true morphology and orientation of the acetabulum is difficult to measure on AP radiographs and we therefore acknowledge that we were limited to measuring the LCEA.

The amount of hip internal rotation was associated with cam morphology presence and size, but limited internal rotation did not precede development of cam morphology. This implies that hip internal rotation decreases as cam morphology develops. It may well be that the rotation is limited by the bony morphology. Several other studies in athletes, such as collegiate football and football players, also showed an association between limited internal rotation and cam morphology [8, 12, 15]. The differences in internal rotation observed between hips with and without cam morphology range from 3 to 6 degrees, depending on cam morphology size. Although this is interesting when trying to understand the etiology and consequences of cam morphology, for clinical purposes this value is below the minimal clinical important difference for measuring internal rotation.

Given the relationship between cam morphology and development of hip OA, there is a need for strategies to prevent the development of cam morphology. Primary prevention would ideally consist of avoiding cam morphology from developing. Given the lower cam morphology prevalence in non-athletes [1, 28], this might be possible by adjusting the loads applied to the athlete’s hip during the second growth spurt. However, to date it is unknown how and when to adjust variables which determine the loads applied to the hip in terms of the exact time frame, frequency, duration and loading patterns. The athletes at highest risk of developing cam morphology could not be identified with the hip parameters studied. The distribution of biomechanical stresses through the proximal femur, as determined by the NSA and EE, were playing a role in the etiology of cam morphology during growth [25]. The risk of cam morphology development in high loading sports must be acknowledged by the clinician, which might also include informing parents of adolescent footballers about these specific potential health disadvantages. These will have to be weighed up against the health benefits of an active lifestyle.

The loss of 40 (45%) of 89 baseline participants might have biased the results. However, the participant characteristics at baseline for the included participants and drop-outs did not differ significantly (Table 2). Of the 40 participants lost to follow-up, 24 rejected the invitation, 11 were not reachable, 4 were playing football abroad and 1 person failed to show up. The longitudinal analyzes might have been underpowered as these comprised only 72 hips without cam morphology at baseline. However, there were almost no absolute differences in the parameters studied between hips that did and did not develop cam morphology, which limits the risk of a type-2 error.

A strength of the study was having three follow-up time-points throughout adolescence and a substantial number of hips having normal morphology at baseline, which is important to study parameters that might precede the development of cam morphology. As only males were included in this study, it is unknown if the results are generalizable to females.

Radiographs instead of 3-dimensional imaging modalities were used, which could have slightly influenced the results. First, it may have led to an underestimation of cam morphology prevalence. Secondly, the NSA, EE and LCEA were only 2-dimensional. However, the correlation between NSA-scores on radiographs and CT is excellent [23]. The NSA on radiographs can be measured optimally on long-leg AP radiographs to optimize the position of the femoral shaft midpoint. The AP radiographs in our study generally showed 5–10 cm below the lesser trochanter, resulting in reliable measurements. The hip internal rotation measurements were performed using a goniometer, which can result in a slight overestimation and measurement errors. Apart from these measurement limitations, physical examination by goniometry is acceptable and reliable for longitudinal studies [21].

For the clinician it is important to understand that this is the first longitudinal study which showed that the studied radiographic and clinical parameters cannot predict cam morphology development. For the clinician and patient, this creates more insight in the etiology of cam morphology and might therefore be useful information in daily practice. Prevention of cam morphology development purely based on the predictive value of specific radiographic or clinical parameter of the hip is not yet possible.

## Conclusion

In conclusion, a varus orientation of the hip, an extended growth plate, and limited hip internal rotation develop simultaneously with cam morphology. None of these hip parameters preceded cam morphology development. These findings underline the importance of the distribution of biomechanical stresses on the growing proximal femur in the etiology of cam morphology.

## Electronic supplementary material

Below is the link to the electronic supplementary material.

Supplementary file1 (DOCX 19 kb)

## Data Availability

For all authors.
