# Supplementary material for: Clinical and radiological hip parameters do not precede, but develop simultaneously with cam morphology: a 5-year follow-up study
Source: Knee Surg Sports Traumatol Arthrosc. 2020 Oct 1;29(5):1401–10. doi: 10.1007/s00167-020-06282-0 (PMC8038954; doi:10.1007/s00167-020-06282-0)
Supplement: Supplementary file 1 — Supplementary file1 (DOCX 19 kb) [file 167_2020_6282_MOESM1_ESM.docx]

**SUPPLEMENTAL TABLES**

| Supplemental Table 1: Cam morphology development during follow-up and baseline preceding parameters (unadjusted associations) | | | |
| --- | --- | --- | --- |
|  | **Development (n = 43)** | **No development (n = 29)** | **P-value** |
| NSA | 133.60° ± 4.78° | 133.18° ± 5.34° | n.s..77 |
| EE | 1.41 ± 0.15 | 1.47 ± 0.19 | n.s..17 |
| LCEA | 26.56° ± 6.07° | 26.25° ± 6.31° | n.s..38 |
| Internal rotation | 28° ± 8° | 28° ± 8° | n.s..50 |
| *Abbreviations: NSA: neck-shaft angle; EE: epiphyseal extension; LCEA: lateral center-edge angle; n.s.: non-significant;*  *Values are expressed as mean ± standard deviation, with n = participants. Associations were not corrected for age and BMI.* | | | |

| Supplemental Table 2: Radiographic and clinical parameters and associated cam morphology presence at baseline | | | | | | | | |
| --- | --- | --- | --- | --- | --- | --- | --- | --- |
|  | **Cam**  **(n = 83)** | **No cam**  **(n = 91)** | **OR, 95%CI, Δ*P*** | ***P*OR, 95%CI, Δ** | **Large cam**  **(n = 21)** | **No large cam**  **(n = 153)** | **OR, 95%CI, Δ*P*** | ***P*OR, 95%CI, Δ** |
| NSA | 129.26° ± 4.74° | 133.31° ± 5.15° | 0.84, 0.79-0.90, 4.1**<.001** | **<0.001** | 128.01° ± 5.01° | 131.84° ± 5.23° | 0.87, 0.78-0.97, 3.8**.009** | **0.01** |
| EE | 1.59 ± 0.19 | 1.49 ± 0.19 | 1.03, 1.01-1.06, 0.10**.008** | **0.01** | 1.66 ± 0.19 | 1.52 ± 0.19 | 1.06, 1.01-1.09, 0.05**<.001** | **<0.001** |
| LCEA | 27.55° ± 6.55° | 27.14° ± 6.73° | -.47 | n.s. | 27.61° ± 7.00° | 27.30° ± 6.60° | -.83 | n.s. |
| Internal rotation | 22° ± 8° | 28° ± 8° | 0.93, 0.90-0.97, 6.**001** | **0.001** | 20° ± 9° | 26° ± 8° | 0.90, 0.83-0.97, 0.01**.005** | **0.01** |
| *Abbreviations; NSA: neck-shaft angle; EE: epiphyseal extension; LCEA: lateral center-edge angle; Abbreviations: CI: confidence interval; EE: epiphyseal extension; LCEA: lateral center-edge angle; n.s.: non-significant; NSA: neck-shaft angle; OR: odds ratio;IR: internal rotation.*  *Values are expressed as mean ± standard deviation, with n = participants and all P-values were corrected for age and BMI. Bolded P-values indicate a statistically significant difference. Due to missing data, the associations in n = 174 hips are presented.* | | | | | | | | |

| Supplemental Table 3: Radiographic and clinical parameters and associated cam morphology presence at 2.5-year follow-up | | | | | | | | |
| --- | --- | --- | --- | --- | --- | --- | --- | --- |
|  | **Cam**  **(n = 81)** | **No cam**  **(n = 35)** | **OR, 95%CI, Δ*P*** | ***P*OR, 95%CI, Δ** | **Large cam**  **(n = 22)** | **No large cam**  **(n = 94)** | **OR, 95%CI, Δ*P*** | ***P*** |
| NSA | 130.59° ± 4.57° | 132.47° ± 5.01° | 0.91, 0.83-0.99, 1.9**.048** | **0.05** | 129.05° ± 4.00° | 131.65° ± 4.81° | 0.88, 0.78-0.99, 2.6**.048** | **0.05** |
| EE | 1.69 ± 0.11 | 1.64 ± 0.09 | 1.05, 1.01-1.09, 0.05**.020** | **0.02** | 1.73 ± 0.12 | 1.66 ± 0.10 | -.05 | n.s. |
| LCEA | 27.37° ± 6.63° | 27.22° ± 6.55° | -.90 | n.s. | 29.29° ± 7.21° | 26.86° ± 6.37° | 1.09, 1.00-1.18, 2.4**.039** | **0.04** |
| Internal rotation | 29° ± 9° | 34° ± 9° | 0.94, 0.89-0.99, 5**.025** | **0.03** | 26° ± 9° | 32° ± 9° | 0.94, 0.89-0.99, 6**.010** | **0.01** |
| *Abbreviations:; CI: confidence interval; NSA: neck-shaft angle; EE: epiphyseal extension; LCEA: lateral center-edge angle; n.s.: non-significant; NSA: neck-shaft angle; OR: odds ratio;*  *Values are expressed as mean ± standard deviation, with n = participants and all P-values were corrected for age and BMI. Bolded P-values indicate a statistically significant difference. Due to missing data, the associations in n = 116 hips are presented.* | | | | | | | | |

| Supplemental Table 4: Radiographic and clinical parameters and associated cam morphology presence at 5-year follow-up | | | | | | | | |
| --- | --- | --- | --- | --- | --- | --- | --- | --- |
|  | **Cam**  **(n=78)** | **No cam**  **(n=20)** | **OR, 95%CI, Δ** | ***P*** | **Large cam**  **(n=25)** | **No large cam (n=73)** | **OR, 95%CI, Δ*P*** | ***P*** |
| NSA | 130.85° ± 4.56° | 133.18° ± 5.35° | 0.85, 0.79-0.99, 2.3 | **0.04** | 128.58° ± 4.18° | 132.27° ± 4.65° | 0.80, 0.71-0.91, 3.7**.001** | **0.001** |
| EE | 1.68 ± 0.11 | 1.66 ± 0.14 | - | n.s. | 1.73 ± 0.10 | 1.65 ± 0.12 | 1.06, 1.01-1.11, 0.08**.024** | **0.02** |
| LCEA | 27.82° ± 6.14° | 29.35° ± 8.26° | - | n.s. | 28.63° ± 6.21° | 27.96° ± 6.77° | -.77 | n.s. |
| Internal rotation | 24° ± 7° | 30° ± 9° | 0.90, 0.84-0.96, 6 | **0.002** | 21° ± 7° | 27° ± 8° | 0.90, 0.82-0.98, 6**.013** | **0.01** |
| *Abbreviations: CI: confidence interval; EE: epiphyseal extension; LCEA: lateral center-edge angle; n.s.: non-significant; NSA: neck-shaft angle; OR: odds ratio;*  *Abbreviations; NSA: neck-shaft angle; EE: epiphyseal extension; LCEA: lateral center-edge angleValues are expressed as mean ± standard deviation, with n = participants and all P-values were corrected for age and BMI. Bolded P-values indicate a statistically significant difference.;*  *Values are expressed as mean ± standard deviation, with n = participants and all P-values were corrected for age and BMI. Bolded P-values indicate a statistically significant difference.* | | | | | | | | |
